# CANDIED: A Pan-Canadian Cohort of Immune Checkpoint Inhibitor-Induced Insulin-Dependent Diabetes Mellitus

**DOI:** 10.3390/cancers14010089

**Published:** 2021-12-24

**Authors:** Thiago P. Muniz, Daniel V. Araujo, Kerry J. Savage, Tina Cheng, Moumita Saha, Xinni Song, Sabrina Gill, Jose G. Monzon, Debjani Grenier, Sofia Genta, Michael J. Allen, Diana P. Arteaga, Samuel D. Saibil, Marcus O. Butler, Anna Spreafico, David Hogg

**Affiliations:** 1 Division of Medical Oncology and Hematology, Princess Margaret Cancer Centre, University of Toronto, University Health Network, Toronto, ON M5S 1Z5, Canada; sofia.genta@uhn.ca (S.G.); m.allen1@uq.edu.au (M.J.A.); darteag@uwo.ca (D.P.A.); sam.saibil@uhn.ca (S.D.S.); marcus.butler@uhn.ca (M.O.B.); anna.spreafico@uhn.ca (A.S.); david.hogg@uhn.ca (D.H.); 2 Hospital de Base, Faculdade de Medicina de Sao Jose do Rio Preto, Sao Jose do Rio Preto 15090-000, Brazil; daniel.araujo@edu.famerp.br; 3 Division of Medical Oncology, Department of Medicine, The University of British Columbia, Vancouver, BC V5Z 1M9, Canada; ksavage@bccancer.bc.ca; 4 Department of Oncology, University of Calgary, Calgary, AB T2N 4N2, Canada; tina.cheng@albertahealthservices.ca (T.C.); jose.monzon@albertahealthservices.ca (J.G.M.); 5 Division of Endocrinology and Metabolism, University of British Columbia, Vancouver, BC V6Z 1Y6, Canada; moumita.saha2@vch.ca (M.S.); sgill@providencehealth.bc.ca (S.G.); 6 The Ottawa Hospital Cancer Centre, University of Ottawa, Ottawa, ON K1H 8L6, Canada; xsong@toh.ca; 7 Department of Medical Oncology, University of Manitoba, Winnipeg, MB R3A 1R9, Canada; dgrenier@cancercare.mb.ca

**Keywords:** immune checkpoint inhibitor, diabetes mellitus, immune-related adverse event, anti-PD1, anti-PD-L1, anti-CTLA4, survival

## Abstract

**Simple Summary:**

Immune checkpoint inhibitor-induced insulin-dependent diabetes mellitus (ICI-induced IDDM) is an emerging form of autoimmune diabetes. We describe the characteristics of 34 patients who developed ICI-induced IDDM across five Canadian cancer centres. We observed that presentation with hyperglycemic crisis is common and that patients treated with combination immunotherapy regimens develop ICI-induced IDDM earlier than those treated with monotherapy. Our results suggest that ICI-induced IDDM is irreversible but is associated with high tumor response rates and prolonged survival. The data generated by this study may help clinicians manage ICI-induced IDDM.

**Abstract:**

Immune checkpoint inhibitor (ICI)-induced insulin-dependent diabetes mellitus (IDDM) is a rare but potentially fatal immune-related adverse event (irAE). In this multicentre retrospective cohort study, we describe the characteristics of ICI-induced IDDM in patients treated across five Canadian cancer centres, as well as their tumor response rates and survival. In 34 patients identified, 25 (74%) were male and 19 (56%) had melanoma. All patients received anti-programed death 1 (anti-PD1) or anti-programmed death ligand-1 (anti-PD-L1)-based therapy. From ICI initiation, median time to onset of IDDM was 2.4 months (95% CI 1.1–3.6). Patients treated with anti-PD1/PD-L1 in combination with an anti-cytotoxic T lymphocyte antigen 4 antibody developed IDDM earlier compared with patients on monotherapy (1.4 vs. 3.9 months, *p* = 0.05). Diabetic ketoacidosis occurred in 21 (62%) patients. Amongst 30 patients evaluable for response, 10 (33%) had a complete response and another 10 (33%) had a partial response. Median overall survival was not reached (95% CI NE; median follow-up 31.7 months). All patients remained insulin-dependent at the end of follow-up. We observed that ICI-induced IDDM is an irreversible irAE and may be associated with a high response rate and prolonged survival.

## 1. Introduction

Endocrine immune-related adverse events (irAEs) occur frequently in cancer patients treated with immune checkpoint inhibitors (ICI) [1,2]. Amongst endocrine irAEs, hypophysitis and thyroiditis are the most frequent. In a metanalysis with data from 6472 patients treated with ICIs in clinical trials, hypophysitis was found to occur more frequently in patients treated with anti-cytotoxic T-lymphocyte antigen 4 (anti-CTLA4) antibodies than in patients treated with anti-programmed death 1 (anti-PD1) or anti-programmed death ligand 1 (anti-PD-L1) monoclonal antibodies (3.8% vs. 1.1%; OR 0.29, 95% CI 0.18–0.49, *p* < 0.0001), whilst hypothyroidism was more common in patients treated with anti-PD1/PD-L1 than in patients treated with anti-CTLA4 antibodies (7% vs. 3.8%; OR 1.89, 95% CI 1.17–3.05, *p* = 0.0089) [3]. Although ICI-related thyroiditis and hypophysitis are well described, ICI-induced insulin-dependent diabetes mellitus (IDDM), a rare and emerging endocrine irAE [4], is less well described.

In a metanalysis of 42 randomized clinical trials, the use of ICIs was not associated with an increased incidence of diabetes, although the risk of hyperglycemia amongst patients using ICIs was increased [5]. Outside the context of clinical trials, the frequency of ICI-induced IDDM has been rising, probably as result of the increasing number of indications of ICIs across several malignancies, such as melanoma, lung carcinoma, renal cell carcinoma, urothelial carcinomas, gastrointestinal malignancies, and squamous cell carcinomas of the head and neck [6,7,8,9,10,11,12,13,14,15]. In the largest series reported to date, the prevalence of ICI-related IDDM was estimated at 0.9% [16].

Due to its rarity, risk factors for ICI-induced IDDM are not well established. Although a correlation with human leukocyte antigen (HLA) subtypes related to classic type 1 diabetes has been described [17], the frequency of islet autoantibodies in patients with ICI-induced IDDM is lower when compared to patients with classic type 1 diabetes (50% vs. 90%) [18]. ICI-induced IDDM is potentially fatal, since it may present with an acute-onset type 1 diabetes phenotype in half of the patients, often with diabetic ketoacidosis (DKA) [19]. 

Although there is an association between the occurrence of irAEs with tumor response [20], multiple metabolic factors may affect the effectiveness of ICI therapy, such as the increased interferon production in diabetic patients with increased body mass index (BMI) [21] and in patients undergoing a ketogenic diet [22]. However, the relationship between ICI-induced IDDM and oncological outcomes, such as response rate and overall survival, has not been studied. 

We aimed to describe the characteristics of ICI-induced IDDM, and to assess the tumor response and survival in a population of ICI-induced IDDM patients. To this end, we conducted a multicentre retrospective analysis across five Canadian academic cancer centres including patients who developed IDDM while on treatment with ICIs. We also present an analysis of concurrent irAEs in a subset of those patients.

## 2. Materials and Methods

### 2.1. Patient Population and Data Collection

Patients ≥ 18 years old from five academic Canadian cancer centres (Princess Margaret Cancer Centre, BC Cancer, Cancer Care Manitoba, The Ottawa Hospital Cancer Centre, and Tom Baker Cancer Centre) who either were on treatment with, or have had exposure to, ICI-based therapy (i.e., monotherapy, combination of two ICI compounds, or combination of ICI with other agents) and developed insulin dependency were included. The diagnosis of insulin dependency was established by the attending physician. Patients with prior non-insulin-dependent diabetes mellitus (NIDDM) and prior pre-diabetes mellitus (pre-DM) who developed insulin dependency while on ICI were also included. Patients who were considered by the investigators to have developed hyperglycemia due to other causes (e.g., steroid-induced hyperglycemia), and patients with prior use of insulin were not included. Data were collected by chart review at each centre using a data collection form developed for this study. The form included primary tumor type, ICI regimen (single agent or combination), type of ICI (anti-PD1/anti-PD-L1, anti-CTLA4, or other), time to development of IDDM from ICI initiation, comorbidities, symptoms, laboratory parameters at IDDM presentation, management of IDDM, tumor response and survival. For the Princess Margaret Cancer Centre (PM) cohort, data on additional irAEs were also collected. Anonymized data were gathered and organized in a spreadsheet by one of the investigators (TPM) for further statistical analysis.

### 2.2. Oncological Outcomes

Response evaluation was recorded according to the assessment of the treating physician registered on the patient chart and/or imaging reports. We did not perform central image review. Objective response rate (ORR) was defined as evaluable patients with a complete response (CR) or a partial response (PR) as determined by their treating physician. Death by any cause without prior progression was recorded as progressive disease (PD). Overall survival (OS) was defined as the time between ICI initiation and death by any cause, or the date when the patient was last known to be alive.

### 2.3. Statistical Analysis

Demographic and clinical data are presented in percentages, means and medians, wherever applicable. Student *t*-test was used to calculate mean differences in glucose and HbA1c levels between specified subgroups. Survival analyses included median OS and median time to ICI-induced IDDM onset. The Kaplan–Meier method was used to generate time-to-event curves and the log-rank method was performed to assess differences between independent groups. A *p*-value < 0.05 (two-tailed) was considered statistically significant. All statistical analyses were performed on SPSS Statistics, version 27.0.

## 3. Results

### 3.1. Patient and ICI Therapy Characteristics

Between July 2016 and April 2021, 34 patients were identified. Median age at ICI-induced IDDM diagnosis was 60.5 years (39–79) and 25 (74%) were male. Nineteen (56%) patients had melanoma (including three patients with uveal and one patient with mucosal melanoma). Renal cell carcinoma (RCC) and non-small cell lung cancer (NSCLC) were the second most frequent malignancies with four (12%) patients each. Thirty (88%) patients were being treated for unresectable/metastatic disease; sixteen (47%) were treated in the first-line setting and four (12%) patients in the adjuvant setting. At the time of ICI-induced IDDM diagnosis, 20 (59%) patients were receiving an anti-PD1/PD-L1 monoclonal antibody in monotherapy, 9 (26%) patients were being treated with an anti-PD1/PD-L1 in combination with an anti-CTLA4 antibody, 3 (9%) were receiving anti-PD1/PD-L1 antibodies combined with other agents, and the remaining 2 (6%) patients were being treated in anti-PD1/PD-L1-based double-blind randomized clinical trials (i.e., anti-PD1/PD-L1 with another agent or placebo). These two patients had not been unblinded by data cut-off. There were no patients treated with anti-CTLA4 antibodies in monotherapy. Only four (12%) patients had a history of ICI treatment in a prior line of therapy.

Hypertension and dyslipidemia were the most frequent comorbidities, occurring in 10 (29%) patients each; prior NIDDM occurred in four (12%) patients and pre-DM occurred in three (9%) patients. Five (15%) patients had a pre-existing autoimmune disease: two had psoriasis, one ulcerative colitis, one Crohn’s disease and one systemic lupus erythematosus. Two (6%) patients had a documented family history of autoimmune disease: one patient had two first-degree relatives with inflammatory bowel disease, and another patient had one first-degree relative with autoimmune diabetes. Three (9%) patients had a history of prior steroid use: two for treatment of ICI-related hepatitis and another for management of spinal cord compression. In these three patients, ICI-induced IDDM occurred after steroid discontinuation, and after (re)exposure to ICIs. Demographic and clinical characteristics at baseline are summarized in Table 1.

### 3.2. ICI-Induced IDDM

**Figure 1:**
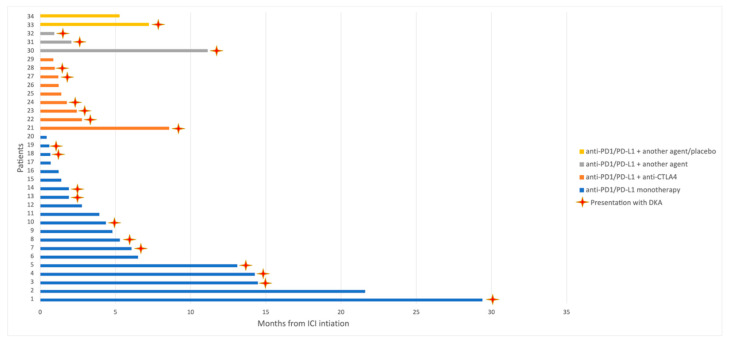
Time to onset of ICI-induced IDDM in 34 patients. Patients are grouped according to ICI treatment modality. CTLA4: cytotoxic T lymphocyte antigen 4; DKA: diabetic ketoacidosis; ICI: immune checkpoint inhibitor; IDDM: insulin-dependent diabetes mellitus; PD1: programmed death 1; PD-L1: programmed death ligand 1.

From ICI initiation, the median time to ICI-induced IDDM onset was 2.4 months (95% CI 1.1–3.6), but time to ICI-induced IDDM varied widely (0.4–29.4 months) (Figure 1). In patients who were being treated with anti-PD1/PD-L1 antibodies in monotherapy, the median time to ICI-induced IDDM onset was 3.9 months (95% CI 0.4–7.4), whilst in patients treated with anti-PD1/PD-L1 and anti-CTLA4 antibodies in combination, the median time to ICI-induced IDDM onset was 1.4 months (95% CI 0.9–1.8) (*p* = 0.05). Median time to ICI-induced IDDM presentation in patients treated with other combinations was 2 months (95% CI 0.2–3.8) and was not significantly different from patients treated with monotherapy (*p* = 0.58) and from patients treated with anti-CTLA4 combinations (*p* = 0.38). When considering all patients treated with combination regimens as one group (anti-CTLA4 or other agents), median time to ICI-induced IDDM onset was not statistically different from patients treated with monotherapy (1.4 vs. 3.9 months, *p* = 0.07). All cases of ICI-induced IDDM occurred while patients were on active treatment.

Glucose level at ICI-induced IDDM diagnosis was available for 32 patients (two patients were diagnosed in outside hospitals and did not have their glucose level registered; one of these patients had a prior diagnosis of NIDDM whilst the other was previously non-diabetic). Mean glucose level was 34.7 μmol/L (SD 15.6) (upper limit of normal (ULN) for random serum glucose = 11.1 μmol/L) [23]. Glucose levels were not statistically different between patients with a prior diagnosis of NIDDM/pre-DM and those without (30.8 μmol/L vs. 35.7 μmol/L; *p* = 0.49), nor between patients treated with monotherapy or combination with anti-CTLA4 agents (33.5 μmol/L vs. 38.4 μmol/L; *p* = 0.48). When accounting for all patients treated with combination immunotherapy (anti-CTLA4 or other agents), there was also no statistical difference compared with patients treated with monotherapy (36.1 μmol/L vs. 33.5 μmol/L; *p* = 0.66).

HbA1c measurements within 30 days of ICI-induced IDDM diagnosis were performed for 19 patients: 10 were receiving anti-PD1/PD-L1 antibodies in monotherapy, 4 were on anti-PD1/PD-L1 and anti-CTLA4 antibodies in combination, 3 were being treated with other anti-PD1/PD-L1-based combinations and 2 were in anti-PD1/PD-L1-based randomized clinical trials. Amongst these 19 patients, 4 had prior pre-DM or NIDDM (one each in a treatment group). Mean HbA1c level was 7.9% (normal < 6.5%) [23] (SD 1.9; mean glucose level was 30.4 μmol/L, SD 9.6) and was not statistically different between patients with prior NIDDM/pre-DM and patients without (8.3% vs. 7.8%, *p* = 0.62). The mean HbA1c levels were 8.8% in patients treated with monotherapy and 7.2% with a combination of anti-PD1/PD-L1 and anti-CTLA4 antibodies (*p* = 0.22). When considering all patients treated with combination regimens as one group, the mean HbA1c levels were 6.9% (*p* = 0.05 when compared with patients treated with monotherapy).

At ICI-induced IDDM presentation, 25 (74%) patients required hospital admission for management of hyperglycemia. In 27 (80%) patients, hyperglycemia occurred acutely, and these patients had a prior routine normal serum glucose level. Accordingly, symptoms of acute hyperglycemia were frequent in our cohort (Table 2) and 21 (62%) presented with DKA (nine of these were receiving combination regimens: six with anti-CTLA4, and three with other agents). There was no significantly increased risk of DKA in patients treated with anti-PD1/PD-L1 and anti-CTLA4 combinations compared with patients treated with anti-PD1/PD-L1 antibodies alone (OR = 1.6; 95% CI 0.3–8.4; *p* = 0.55). In seven (20%) patients, hyperglycemia development was insidious and was initially managed as NIDDM; in these cases, however, glucose levels were poorly controlled despite the optimization of oral anti-diabetic agents, and adequate diabetes control was only achieved after the introduction of insulin replacement therapy. C-peptide was measured in 17 patients and seven patients had undetectable levels; another six patients had detectable but decreased C-peptide levels, and four had normal levels. Anti-glutamic acid decarboxylase 65 was available for 11 patients, and five had increased levels. Only one patient had an assessment of anti-insulin antibody, and it was negative. None of the patients in our cohort had anti-islet cell or anti-zinc transporter 8 measurements.

All patients received insulin replacement therapy. Additionally, four (12%) patients received immunosuppressive therapy for management of ICI-induced IDDM. One patient was treated with infliximab, and another patient received steroids and infliximab exclusively for management of ICI-induced IDDM. The former had an esophagogastric adenocarcinoma and had PD as best response to immunotherapy, and the latter remained on surveillance after achieving a complete metabolic response of metastatic melanoma. In both cases, infliximab was administered based on guideline recommendations for the management of steroid-refractory irAEs [24] (steroids were not used in the first case due to concerns of worsening hyperglycemia). The other two patients were treated with steroids for concurrent irAEs (bone marrow hypoplasia and increased lipase). One patient (3%) died 24 h after presenting to the emergency department with DKA. Following ICI-induced IDDM onset, 19 (56%) patients discontinued immunotherapy. At the end of the observation period, all patients were insulin dependent.

### 3.3. Response Assessment and Overall Survival

Thirty patients were evaluable for response assessments; 10 (33%) patients had a CR and another 10 (33%) patients had PR (ORR = 66%). This response rate was largely driven by the melanoma cohort, in which 14 of the 18 evaluable patients (77%) presented a tumor response. Three (10%) patients had stable disease (SD) as best response and the remaining seven (26%) patients had PD. Response assessment according to primary tumor type is presented in Supplementary Figure S1.

Amongst the 19 patients who discontinued immunotherapy after ICI-induced IDDM onset, one was rechallenged with ICI upon disease progression following a PR of metastatic melanoma. This patient had no further response to treatment and died due to PD. Another four patients died due to PD without further rechallenge: one had NSCLC, one had esophagogastric adenocarcinoma (these two patients had PD as best response to ICI), and the remaining two patients had melanoma. One had cutaneous melanoma and developed hemorrhagic brain metastases after 6 months of interrupting adjuvant therapy, and the other had metastatic uveal melanoma that did not respond to treatment. Another patient with metastatic melanoma died due to complications of immune-related bone marrow hypoplasia without documented PD. The remaining 13 patients who discontinued treatment were alive with sustained complete or partial responses, or without relapse (three patients treated in the adjuvant setting), at the end of the follow-up period.

**Figure 2:**
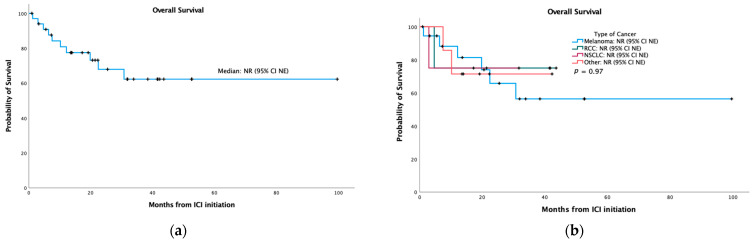
Kaplan–Meier curves for overall survival: (**a**) in the overall population; (**b**) stratified by primary tumor type. ICI: immune checkpoint inhibitor; NE: non-estimable; NR: not reached; NSCLC: non-small cell lung cancer; RCC: renal cell carcinoma.

After a median follow-up of 31.7 months (range 0.9–99.6), median overall survival was not reached (95% CI NE) in the overall population (Figure 2a) or in any tumor subgroup (Figure 2b).

### 3.4. Princess Margaret Cancer Centre Cohort: Additional irAEs

In fifteen (44%) patients treated at PM, we analyzed the occurrence of additional irAEs (since ICI initiation until date of last follow up). The baseline characteristics of these patients were not significantly different when compared to patients treated in other centres (Supplementary Table S1).

Table 3 shows the frequency of additional irAEs according to the Common Terminology Criteria for Adverse Events (CTCAE) version 5.0 [25]. All patients had at least one additional irAE; increase in aminotransferases was the most frequent, with one patient presenting a grade 3 increase in AST. Two patients had vitiligo-like skin depigmentation. An increase in amylase and lipase was documented for one patient (grade 2 and grade 3, respectively) without clinical pancreatitis. Another patient had laboratory-proven steatorrhea that improved with exocrine pancreatic enzyme replacement therapy.

## 4. Discussion

To the best of our knowledge, this is the largest series of patients with ICI-induced IDDM published to date. Previously, Stamatouli et al. published data on 27 patients from two different cancer centres in the United States [16]. Our series is representative of the current approved indications for ICIs and similarly to other published series [4,7,16,19], we observed a preponderance of patients with melanoma.

All our patients were receiving anti-PD1/PD-L1 monoclonal antibodies, either as monotherapy or in combination with different agents, and we observed that combination with anti-CTLA4 antibodies may accelerate the onset of ICI-induced IDDM. In patients with type 1 diabetes, upregulation of PD-L1 in pancreatic β-cells occurs as a mechanism to attenuate the immune assault in the early stages of type 1 diabetes [26]. Moreover, patients with latent autoimmune diabetes of adulthood (LADA) who carry the G6230A variant of the *CTLA4* gene have a more precipitous loss of β-cell function and more rapidly become insulin dependent [27]. Taken together, these data suggest that blockade of two non-redundant immune checkpoint pathways (PD-L1 and CTLA-4) may accelerate the immune infiltration into pancreatic islets, and we hypothesize that blockade of PD-L1 is a key factor for the development of ICI-induced IDDM in predisposed individuals.

In our cohort, we observed that five patients had an underlying autoimmune disease, of which some possessed well described predisposing HLA subtypes that overlapped with those of autoimmune diabetes. For example, HLA-DRB1*03 and HLA-DRB1*04, both previously associated with LADA [28], also confer an increased risk for ulcerative colitis (OR = 3.6) and Crohn’s disease (OR = 3.9), respectively [29]. HLA-DRB1*03:01 was associated with the development of type 1 diabetes of the youth in a Pakistani population [30] and is also associated with increased susceptibility to systemic lupus erythematosus with the production of anti-Ro and anti-La antibodies [31]. Some of these alleles have been described in patients with ICI-induced IDDM in other studies [16,17]. Our data are limited by the lack of HLA characterization, but we hypothesize that there may be a subgroup of patients with autoimmune diseases that are at increased risk of ICI-induced IDDM, and further characterization of HLA subtypes may aid in the identification of such patients. 

However, as observed by Stamatouli et al. [16] and in our cohort, most patients with ICI-induced IDDM do not have a prior autoimmune disease. As is the case of type 1 diabetes, a combination of environmental factors and genetic susceptibility may be necessary for ICI-induced IDDM onset. For instance, infection by cytomegalovirus (CMV) is a potential trigger for type 1 diabetes [32] and a recent study demonstrated that CMV reactivation may be implicated in some cases of ICI-related hepatitis [33]. Whether occult viral infections may lead to the development of ICI-induced IDDM in predisposed individuals remains to be explored.

Most of our patients presented with overt hyperglycemia and disproportionately low HbA1c levels [34,35]. This suggests that the immune assault to the pancreas initiates within the first cycles of immunotherapy, leading to rapid β-cell exhaustion, as indicated by low or undetectable C-peptide and presentation with DKA. Although DKA has a low mortality rate in young patients with classic type 1 diabetes, the mortality rate in elderly patients with comorbidities may be higher than 5% [36]. Prompt recognition of evolving hyperglycemia is necessary for patients on treatment with ICI, but reliable markers for its detection are lacking in clinical practice. A small prospective observational study reported a small but statistically significant increase in HbA1c in patients treated with ICIs [37]. However, the value of regular HbA1c monitoring in patients treated with ICIs, as suggested by Akturk and Michels [38], should be assessed prospectively. 

Although ICI-induced IDDM usually presents early during treatment, as observed by us and others [4,16,19,39], some patients develop insulin dependency as late as after 30 months of exposure to ICI, without DKA and with no expression of autoantibodies commonly found in patients with other types of autoimmune diabetes. This indicates that ICI-induced IDDM is a heterogeneous entity that mimics the wide spectrum of autoimmune diabetes, from fulminant type 1 diabetes, more frequently seen in the Asian population [40], to LADA, which is frequently misdiagnosed as difficult-to-treat type 2 diabetes [41].

Non-endocrine irAEs are typically managed with steroids and other immunosuppressants, such as infliximab, when they become steroid refractory. Endocrine irAEs, however, are managed with the aim to control symptomatology (e.g., ICI-related hyperthyroidism) and with hormone replacement therapy [24]. Akin to other endocrine irAEs, ICI-induced IDDM was permanent, even in patients treated with steroids and infliximab. Trinh et al. reported one case of presumably ICI-induced IDDM that reversed after the use of infliximab for ICI-related arthritis [42]; however, the patient had evidence of peripheral insulin resistance, and infliximab may have upregulated glucose transporter mechanisms, thus contributing to the reversal of hyperglycemia [43]. Taken together, there is no evidence to support the use of steroids and other immunosuppressants for the management of ICI-induced IDDM, and our data do not suggest any benefit from such an approach. Therefore, patients who develop ICI-induced IDDM should be managed according to current guidelines for IDDM of other causes [44,45].

The high response rate and prolonged survival in our cohort are consistent with previous studies demonstrating improved oncological outcomes in patients who develop irAEs. While this correlation is not absolute and may be influenced by environmental factors [21,22,46], the survival benefit is usually maintained despite the discontinuation of ICIs in a proportion of patients [20]. Indeed, all patients in the PM cohort experienced other irAEs, including two patients with vitiligo, which is related to tumor response and survival in patients with metastatic melanoma [47]. The high survival rate in patients with ICI-induced IDDM implies that these patients will require long-term management of hyperglycemia to delay the development of chronic complications of diabetes [34]. The assessment of the long-term consequences of ICI-induced IDDM, including its impact on quality of life, will require further follow up. 

Our study has limitations, including its retrospective design and missing data in a proportion of patients. In addition, the lack of HLA genotyping in our population prevents us from formulating more consistent hypotheses on the pathophysiology of ICI-induced IDDM. We also did not have data on BMI, which precludes any inference on its influence in the development of ICI-induced IDDM. It should be noted, however, that in a prior study [16] the average BMI was not significantly high (26.07 Kg/m^2^) and was not associated with the occurrence of autoantibodies. Nonetheless, we show that ICI-induced IDDM usually occurs acutely and may be potentially fatal. In addition, our data suggest that ICI-induced IDDM is triggered by blockade of the PD1/PD-L1 axis. Our results may raise the awareness of treating physicians for the development of insulin dependence in patients on ICI therapy, as well as provide guidance on the management of this emerging irAE and facilitate cancer-related treatment decisions.

## 5. Conclusions

In summary, ICI-induced IDDM is an emerging type of autoimmune diabetes that most frequently manifests near the beginning of ICI therapy with hyperglycemic crisis, mimicking acute-onset type 1 diabetes. We stress, however, that it may present at any time during ICI therapy, with a heterogenous clinical spectrum that resembles other types of autoimmune diabetes. Further studies are necessary to better identify patients at risk to develop ICI-induced IDDM and develop strategies for the early detection of IDDM. In our cohort, the occurrence of ICI-induced IDDM was associated with high response rates and improved survival. Continuous follow up to characterize the long-term complications of ICI-induced IDDM is warranted.

## Figures and Tables

**Table 1:** Demographic and baseline characteristics of patients with ICI-induced IDDM.

Population	34 Patients
Median Age (Range)	60.5 (39–79)
Sex	*n* (%)
Male	25 (74%)
Female	9 (26%)
Primary Tumor	*n* (%)
Melanoma	19 (56%)
Renal cell carcinoma	4 (12%)
Non-small cell lung cancer	4 (12%)
Other ^1^	7 (20%)
Comorbidities	*n* (%)
Hypertension	10 (29%)
Dyslipidemia	10 (29%)
Obesity	3 (9%)
NIDDM	3 (9%)
Pre-DM	4 (12%)
Hypothyroidism	5 (15%)
Autoimmune disease ^2^	5 (15%)
ICI Regimen	*n* (%)
Anti-PD1/PD-L1 monotherapy	20 (59%)
Anti-PD1/PD-L1 + anti-CTLA4	9 (26%)
Anti-PD1/PD-L1 + another agent	3 (9%)
Anti-PD1/PD-L1 + another agent/placebo	2 (6%)
Line of Therapy	*n* (%)
First	16 (47%)
Second	7 (21%)
Third or beyond	6 (17%)
Adjuvant	4 (12%)
Consolidation	1 (3%)

^1^: Other tumor types include head and neck squamous cell carcinoma (2), gastroesophageal junction adenocarcinoma, urothelial carcinoma, breast carcinoma, and hepatocellular carcinoma (1 each); ^2^: autoimmune diseases were psoriasis (2), Crohn’s disease, ulcerative colitis and systemic lupus erythematosus (1 each). CTLA4: cytotoxic T-lymphocyte antigen 4; DM: diabetes mellitus; IDDM: insulin-dependent diabetes mellitus; NIDDM: non-insulin-dependent diabetes mellitus; PD1: programmed death 1; PD-L1: programmed death ligand 1.

**Table 2:** Frequency of symptoms at presentation of ICI-induced IDDM in 34 patients.

Symptom	*n* (%)
Polydipsia	19 (56%)
Polyuria	14 (41%)
Dehydration	9 (26%)
Weight loss	6 (17%)
Abdominal pain	4 (12%)
Confusion	4 (12%)

ICI: immune checkpoint inhibitor; IDDM: insulin-dependent diabetes mellitus.

**Table 3:** Additional irAEs in the Princess Margaret Cancer Centre cohort.

Immune-Related Adverse Events	All Grades ^a^	G1	G2	G3	G4
AST increased	7	6	0	1	0
ALT increased	8	5	3	0	0
Troponin increased	1	1	0	0	0
CPK increased	1	0	0	0	1
Pancreatic enzymes decreased	1	0	1	0	0
Amylase increased	1	0	1	0	0
Lipase increased	1	0	0	1	0
Hypothyroidism	6	0	6	0	0
Hyperthyroidism	3	2	1	0	0
Diarrhea	2	0	1	1	0
Colitis	2	0	2	0	0
Rash	3	1	2	0	0
Pruritus	1	1	0	0	0
Vitiligo-like skin depigmentation	2	0	2	0	0
Hyperkeratosis	1	0	0	1	0
Guillain–Barré syndrome	1	0	1	0	0
Total	41	16	20	4	1

^a^: Grades according to the Common Terminology Criteria for Adverse Events, version 5.0. ALT: alanine aminotransferase; AST: aspartate aminotransferase; CPK: creatine phosphokinase; irAEs: immune-related adverse events.

## Data Availability

The data presented in this study are available within this article.

## Supplementary Materials

The following are available online at https://www.mdpi.com/article/10.3390/cancers14010089/s1, Figure S1: Response assessment according to primary tumor type, Table S1: Comparison of baseline characteristics between patients with ICI-induced IDDM treated at Princess Margaret and other cancer centres.
