# Supplementary material for: CANDIED: A Pan-Canadian Cohort of Immune Checkpoint Inhibitor-Induced Insulin-Dependent Diabetes Mellitus
Source: Cancers (Basel). 2021 Dec 24;14(1):89. doi: 10.3390/cancers14010089 (PMC8750429; doi:10.3390/cancers14010089)
Supplement: Supplementary file 1 [file cancers-14-00089-s001.zip › cancers-1468366-supplementary.pdf]

cancers

MDPI

Supplementary Materials

CANDIED: A Pan-Canadian Cohort of Immune Checkpoint Inhibitor-Induced Insulin-Dependent Diabetes Mellitus

Thiago P. Muniz, Daniel V. Araujo, Kerry J. Savage, Tina Cheng, Moumita Saha, Xinni Song, Sabrina Gill, Jose G. Monzon, Debjani Grenier, Sofia Genta, Michael J. Allen, Diana P. Arteaga, Samuel D. Saibil, Marcus O.Butler, Anna Spreafico and David Hogg

Copyright: © 2021 by the authors. Licensee MDPI, Basel, Switzerland. This article is an open access article distributed under the terms and conditions of the Creative Commons Attribution (CC BY) license (https://creativecommons.org/licenses/by/4.0/).

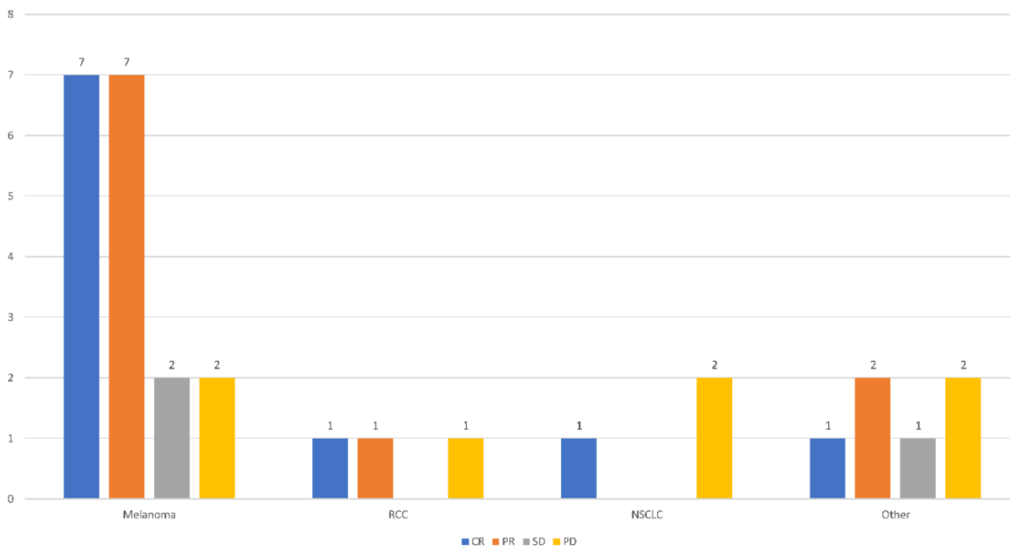

Figure S1. Response assessment according to primary tumor type. CR: Complete response; PR: Partial response; SD: Stable disease; PD: Progressive disease; RCC: Renal cell carcinoma; NSCLC: Non-small cell lung cancer.

Table S1. Comparison of baseline characteristics between patients with ICI-induced IDDM treated at Princess Margaret and other cancer centers.

	**Princess Margaret (N=15)**	**Other centers (N=19)**	***p* value**
Median age (range)	56 (48-77)	61 (39-79)	1.00
Mean glucose (SD)	37.1 (16.5)	32.9 (15.2)	0.45
Mean HbA1c (SD)	6.9 (1.3)	8.9 (2)	0.02
Sex			
Male	11 (73%)	14 (74%)	0.98
Female	4 (27%)	5 (26%)	
Primary tumor			
Melanoma	8 (53%)	11 (58%)	0.79
Renal cell carcinoma	2 (13%)	2 (10%)	0.80
Non-small cell lung cancer	1 (7%)	3 (16%)	0.41
Other	4 (27%)	3 (16%)	0.43
ICI regimen			
Anti-PD1/PD-L1 single agent	8 (53%)	12 (64%)	0.56
Anti-PD1/PD-L1 + Anti-CTLA4	4 (27%)	5 (26%)	0.98
Anti-PD1/PD-L1 + another agent	1 (7%)	2 (10%)	0.69
Anti-PD1/PD-L1 + another agent/placebo	2 (13%)	0	0.10

CTLA4: Cytotoxic T-lymphocyte antigen 4; ICI: Immune checkpoint inhibitor; IDDM: Insulin-dependent diabetes mellitus; PD1: Programmed death 1; PD-L1: programmed death ligand 1; SD: Standard deviation.
